# Retrospective Review of Loop Electrosurgical Excision Procedure (LEEP) Outcomes at a Tertiary Hospital in Zambia

**DOI:** 10.1155/2020/1920218

**Published:** 2020-08-19

**Authors:** Nancy Kasongo, Chiza Kasungu, Nixon Miyoba, Herbert T. Nyirenda, Muleta Kumoyo

**Affiliations:** ^1^School of Medicine, The Copperbelt University, Ndola 10101, Zambia; ^2^University of Zambia, Lusaka 10101, Zambia; ^3^Department of Obstetrics and Gynecology, Ndola Teaching Hospital, Ndola 10101, Zambia

## Abstract

There is a lack of knowledge on the histologic outcomes of loop electrosurgical excision procedure (LEEP) biopsies in the diagnosis, treatment, and prevention of cervical cancer in Zambia. This study determined the outcomes of LEEP biopsies and associated factors at a tertiary hospital. We conducted a retrospective chart review of patients evaluated at a tertiary hospital cervical cancer screening centre. From the database, we identified patients who underwent LEEP between January 2015 and June 2018. We extracted demographic data, HIV data, and LEEP biopsy results. A *P* value less than 0.05 was considered statistically significant. 137 charts were identified, and 114 were included in the final analysis. 23 were excluded for missing histology. The mean age of participants was 36.3 ± 9.6. Histology outcomes revealed that 37% had cervicitis, while CIN 1, 2, and 3 contributed to 27%, 14%, and 3%, respectively. Squamous cell cancer was present in 8% (age groups 35–49) and was three times higher (13%) in HIV-positive compared to HIV-negative participants (3.8%). Normal histology accounted for 11%. Increasing age (*P*=0.029), less than tertiary education (*P*=0.0011), and being married (*P*=0.017) increased the chances of having cancer in the chi-square analysis, while single women had lower odds of having CIN 1 (OR = 0.012) in the multinomial logistic regression. There is a need for increased cervical cancer screening and training in precancer treatment and holistic consideration of other factors like age in addition to the positive VIA test in advising patients on treatment options.

## 1. Introduction

Cervical cancer (CC) is the second most common cancer in women after breast cancer in developing countries and is a cause of over 266,000 deaths globally with nine out of ten deaths occurring in the least-developed areas [1–4]. The incidence of cervical cancer worldwide varies from as low as 3.8 per 100,000 in western Asia to as high as 34.8 per 100,000 in sub-Saharan Africa (SSA), respectively [1, 5]. In Zambia, the incidence of CC is 58.0 per 100,000, the second highest incidence in the world [2, 3, 6], while the mortality rate is at 36.2 per 100,000. Cervical cancer is a prominent cause of mortality and morbidity in Zambia [2, 6].

The development of CC is closely related to human papilloma virus (HPV), where types 16 and 18 (high-risk types) have been implicated in about 70% of cervical cancer cases around the globe. Zambia has reported a very high prevalence of 21.6% for both HPV 16 and 18 [2]. About 2% of women globally have advanced precancerous lesions with the rate being five times higher in human immunodeficiency virus- (HIV-) positive women [7]. With an adult HIV prevalence rate of 16% in Zambia [2], many women have a high risk to developing CC.

The Cervical Cancer Prevention Program in Zambia (CCPPZ) was established in 2006 through joint efforts of the Centre for Infectious Disease Research in Zambia (CIDRZ) and the Zambian Ministry of Health [8]. The CCPPZ is blended into the already functioning HIV/AIDS care and treatment facility, and screening is done using visual inspection with acetic acid (VIA) enhanced by digital cervicography (DC) [2, 3]. Zambia adopted the “screen and treat” approach formulated by the World health organization (WHO) to reduce CC mortality. Using the approach, a treatment decision which is based on a screening test and not biopsy confirmed CIN 2+ and treatment is prompt on the same day after a screening examination [7]. The key treatments available in Zambia are cryotherapy and LEEP [1].

The literature on histopathologic outcomes in patients after treatment with LEEP shows that CIN 1 accounts for 21.9%, CIN 2 or 3 (72.5%), and CC (5.6%) [9]. Furthermore, a study assessing development, implementation, and evaluation of the Cervical Cancer Prevention Program in Zambia found that among women undergoing LEEP, HIV women reported CC cases three times higher than HIV-negative women [6].

There has been controversy over the factors that are associated with CIN and CC, and some authors who assessed age, marital status, education, income, being on antiretroviral therapy, life-time partners, and sexual debut found that these were not in any way associated with CIN or CC [3]. However, other authors have found that not being on antiretroviral therapy was marginally associated with CIN or CC (*P* value = 0.005) [10]. The aim of this study was to determine the histologic outcomes of LEEP and associated factors at Kitwe Teaching Hospital which was achieved.

## 2. Methods

This was a retrospective cohort study conducted at the Kitwe Teaching Hospital Cervical Cancer Screening Centre. The screening centre was established in 2013 and caters for Kitwe and the surrounding areas. Medical records of patients who underwent LEEP from January 2015 to June 2018 were retrieved from the CCPZ database using a data extraction sheet, while histology results were obtained from the histopathology department register and matched with patients using the unique hospital patient identification number. The study collected data on age, education status, marital status, HIV status, number of sexual partners, and age of the sexual debut.

Cervical cancer screening at the centre was done using visual inspection with acetic acid (VIA) enhanced by digital cervicography (DC) by trained nurses. The treatment option offered for suspected CIN was LEEP which was performed by trained medical doctors. The eligibility criteria for LEEP included a lesion covering more than 75% of the ectocervix or extending into the endocervical path. LEEP was done under the screen and treat concept in all the patients presented in this study. Counselling on risks and benefits of LEEP was done before the procedure. Biopsies collected during the procedure were examined at the hospital laboratory or a private laboratory. Patients were reviewed after 6 weeks with results of biopsy but could be seen much earlier if patients experience bleeding or any complications due to the procedure. Patients with confirmed invasive cancer were then referred to gynecologists. The study included all the women that attended cervical screening at the cervical clinic and had a positive result with VIA and underwent LEEP. Patients with missing histology results and those who underwent cryotherapy for treatment were excluded from the study.

A total of 137 records were identified as those of women who underwent LEEP at the centre: 40 in 2015, 26 in 2016, 39 in 2017, and 32 between January and June 2018. Twenty-three records were excluded as they lack histology results, leaving a sum of 114 records that were analysed. Forty-four records where incomplete (some of the demographic data that the study was collecting were missing); however, they were included in the final analysis because the records met the inclusion criteria. Some of the incomplete records were missing at the study site, whereas others could not be assessed as the women were referrals from nearby towns were they were initially screened.

Data collected through extraction sheets were entered into Microsoft Excel 2016 version and analysed using the Statistical Package for Social Science (SPSS) version 23. The data were presented as the mean ± standard deviation (SD), frequency (*n*) and percentage (%), or odds ratio (OR) and a 95% confidence interval (CI), as appropriate. Factors associated with LEEP were analysed using a chi-squared and multinomial logistic regression analysis.

The authority to conduct the research was sought from the Research Ethics Committee based at the Tropical Disease Research Centre (TDRC), Ndola, through the Copperbelt University School of Medicine authorities. Permission was also sought from Kitwe Teaching hospital to access patient records as this was a retrospective study. To protect patient identity, the CCPZ and hospital file numbers were removed, and records were reassigned a unique identification number.

## 3. Results and Discussion

### 3.1. Results

#### 3.1.1. Characteristics of Study Participants

The demographic features of women undergoing LEEP are shown in Table 1. The mean age of women undergoing LEEP was 36.3 ± 9.6. There were more patients in the age group 40–49 in both the no CIN/CIN 1 and CIN 2+ groups. Patients whose LEEP biopsy tested positive for CIN 2+ were less educated than those whose histology grade was low. Majority of the study participants were married (58/70). The mean age of the sexual debut was 18.6 ± 2.8, while the mean number of sexual partners was 3 ± 2.5. Among the patients undergoing LEEP, almost half were HIV positive (47.4%).

#### 3.1.2. Histology Outcomes

**Figure 1:**
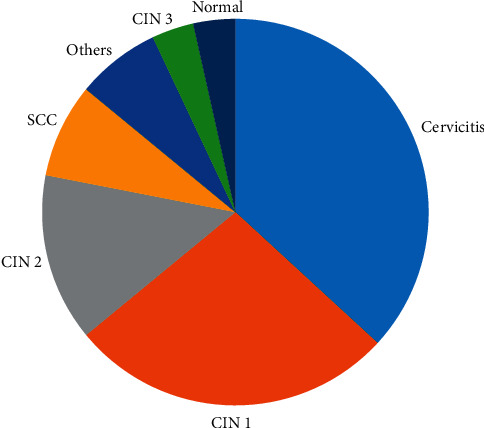
Histology outcomes of women who had LEEP from January 2015 to June 2018. CIN, cervical intraepithelial neoplasia; SCC, squamous cell carcinoma.

As depicted in Figure 1, the histology outcomes reveal that 37% of the women undergoing LEEP had cervicitis. CIN 1 accounted for 27%, while 29 (25%) had CIN 2+, in particular, 14% had CIN 2, 3% had CIN 3, and 8% had SCC. Squamous cell carcinoma (SCC) was the only reported cancer in this group, while the number of subjects in whom no abnormality was found was 4%. Other cervical conditions described in histology findings were endometriosis, cervical polyp, and early signs of viral colonization in cervical cells accounting for 7%.

#### 3.1.3. Factors Associated with LEEP Outcomes

**Figure 2:**
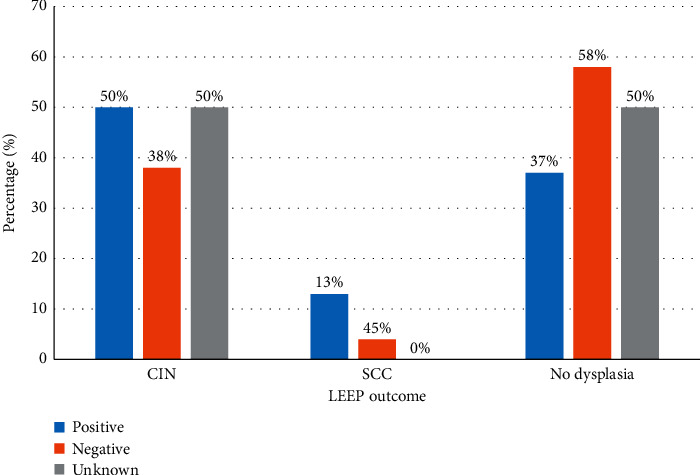
Percentage distribution of HIV status (positive/negative) by the LEEP outcome. HIV, human immunodeficiency virus; CIN, cervical intraepithelial neoplasia; SCC, squamous cell carcinoma.

Chi-square analysis was done to establish the association between selected factors (HIV status, age, age of sexual debut, marital status, education, and number of sexual partners) and LEEP outcomes. Chi-square test revealed that increasing age (*P*=0.029), less than tertiary education (*P*=0.0011), and having being married before (*P*=0.017) increased the chances of having cervical cancer. Amongst the HIV-negative and positive groups, squamous cell cancer was three times higher (13%) in HIV positive compared to HIV negative (3.8%) as shown in Figure 2. Multinomial logistic regression analysis included age, education, marital status, sexual debut, number of sexual partners, and HIV status. Results suggest that being unmarried reduced the chances of having CIN 1 (*P*=0.017) OR (0.12).

## 4. Discussion

This study shows a prevalence of cervical cancer of 8%. The current finding is higher than what was reported in a recent study that was conducted in Zambia among HIV-positive women in which the rate of CC was 1.9% [3]. A similar study in Thailand on LEEP outcomes found the prevalence of CC to be 5.4% [9]. Among the HIV-positive and negative groups, SCC was three times higher in HIV positive than HIV negative. This result is identical to the findings of a study conducted in Zambia on cervical cancer prevention services which found that ICC was 3.4 times more common in HIV positive [6]. One theory explaining why HIV-positive people are more prone to ICC is that the immunosuppression that is as a result of HIV increases the liability to HPV along with infection with numerous strains and persistent HPV and ultimately fails to eliminate HPV due to a weakened immune system which is responsible for the rise in incidence and recurrence of CC in HIV [3, 4].

Results of this study suggest that the prevalence of CIN 1 lesions was 27%. Other studies have reported a prevalence of 15.5% and 42.7% in studies conducted in Thailand and Zambia [3, 9]. The observed differences could be due to differences in HIV prevalence, as HIV increases the risk of CIN and CC [7]. We had very few women with CIN 3 in our study (3%) as compared to 64% in another study [9]. A possible explanation for this would be that women in this study were screened late when the cervical disease has progressed from precancer (CIN) to cancer.

The proportion of women undergoing LEEP in the study who had low-grade LEEP biopsy findings was 75%. In contrast to our study findings, a study on LEEP outcomes in Thailand that utilized cytology-based screening reported a much lower prevalence of 21.9% [9]. The high false-positive rate in our study could have been attributed to the use of VIA alone for cervical cancer screening in the current study setting. Visual inspection with acetic acid has been shown to have a low sensitivity and specificity when compared to other screening tests [11]. Furthermore, the literature indicates that HPV DNA testing has the highest sensitivity and specificity, followed by cytology-based screening tests [11]. Thus, when screening for CC with VIA in resource-limited settings, utilizing a second test could prevent over treatment with LEEP in patients without CIN or cancer. Rapid point-of-care HPV DNA assays that are affordable if made accessible are a great option because they do not require expertise or expensive equipment and can be incorporated into routine screening of CC with VIA with ease in low-resource settings. Another explanation for this finding could be that young patients (less than 30 years) who accounted for 28.1% of 114 patients were treated with LEEP when most of the literature indicates that in most HPV-related CIN under the age of 30, spontaneous regression is expected and conservative treatment with follow-up is recommended [12]. This finding could also indicate a need for more training in precancer treatment and holistic consideration of other factors like age in addition to a positive VIA test in advising patients on treatment options.

According to the WHO guidelines for screening and treatment of precancerous lesions for cervical cancer prevention, screening should be done one year after LEEP to exclude cancer [7]. A number of women undergoing LEEP in the current study where referred from different towns for LEEP treatment at the study site. During the study period, the CCPPZ records of the patient from other sites were not synced to a central hub creating a hurdle in accessing information needed for treatment and follow-up of such patients after LEEP. However, this obstacle has become obsolete in the Cervical Cancer Prevention Program in Zambia where a national cervical cancer database was developed and launched in the late 2019. The database called “SmartCerv” was financed from multiple grants and will enable direct patient data collection, secure storage, and aggregate reporting of information stemming from the screening and treatment program [13]. The system can be installed on a smart phone, tablet, or computer, making it extremely easy to retrieve and capture patient information for CC because patients undergoing LEEP are mobile and not all screening centres are capable of performing LEEP. Follow-up of patients who undergo LEEP is very important as the procedure is not only diagnostic but is also a treatment modality. LEEP follow-ups are an important population in whom development of CC can be curbed and other treatments are instituted provided they are followed up periodically.

Regarding factors associated with LEEP outcomes, using the bivariate analysis, age, education, and marital status were shown to increase the CC risk, while in the multinomial logistic regression, unmarried status reduced the odds of having CIN 1. Our study outcomes are in contrast with the findings of a cross-sectional study that described the magnitude of CIN and CC in Zambia which found that in the multinomial logistic regression, none of the demographic factors were associated with CIN or cervical cancer [3].

One obstacle in our study was the loss of records due to poor record keeping at the study site as there were unforeseen hardware failure on the computer used for the entry of LEEP records. Other records could not be assessed because patients were referrals from other centres where they were initially screened, and we could not also access LEEP biopsy results when the sample was taken to a private laboratory. Further, the study used data from medical records captured in an established database, hence was limited to the indicators captured. In addition, demographic data (excluding age and HIV status) were available in only 70 patients which limited the ability to identify risk factors for CIN or cancer. Few independent variables were used due to poor record keeping. Finally, our prevalence of SCC and CIN could be underestimated because we only used VIA to screen for CC. The study was also able to identify cervicitis as a cause of 37% of the positive VIAs. Further research is needed to define the etiology of cervicitis among this group as the literature has shown a positive association between developing high-grade lesions and increasing levels of inflammation of the cervix [14]. Zambia has reported high rates of oncogenic HPV 16 and 18 [2]. It is unclear what percentage of chronic cervicitis is attributed to oncogenic HPV and whether this contributes to our high cervical cancer rates. Future studies could also prospectively assess LEEP outcomes at one-year follow-up.

## 5. Conclusions

Utilizing VIA and LEEP biopsy, this study was able to highlight a high disease burden of cervical cancer of 8% when compared to other literature studies. The see and treat approach with VIA as a screening test and LEEP as a form of treatment as in this study is a great opportunity to reduce mortality and morbidity due to CC. However, the findings of this study suggest a need for more training in precancer treatment and holistic consideration of other factors like age in addition to the positive VIA test in advising patients on treatment options due to the high false-positive rate (75%). The study also emphasizes a need for research to define the etiology of cervicitis in patients undergoing LEEP as this contributed to 37% of histology findings and is essentially treatable.

## Figures and Tables

**Table 1:** Percentage distribution of characteristics of women undergoing LEEP from January 2015 to June 2018 (no CIN/CIN 1 vs CIN 2+).

		No CIN/CIN 1	CIN 2+	Total
Age	20–24	14	0	14
	25–29	14	4	18
	30–34	17	2	19
	35–39	11	4	15
	40–49	23	15	38
	Above 50	6	4	10
Total		85	29	114

Education level	No formal education	0	2	2
	Less than high school	12	5	17
	High school	14	6	20
	Tertiary	29	2	31
Total		55	15	70

Marital status	Never married	11	1	12
	Ever married	44	14	58
Total		55	15	70

Sexual debut	Less than 14	1	2	3
	Above 15	54	13	67
Total		55	15	70

Number of sexual partners	1–4	48	12	60
	5–15	7	3	10
Total		55	15	70

HIV status	Positive	34	20	54
	Negative	45	7	52
	Unknown	6	2	8
Total		85	29	114

## Data Availability

All data pertaining to the study are presented in the manuscript.
